# FGF10 Is Required for Circumvallate Papilla Morphogenesis by Maintaining *Lgr5* Activity

**DOI:** 10.3389/fphys.2018.01192

**Published:** 2018-08-28

**Authors:** Sushan Zhang, Hyuk Su Choi, Han-Sung Jung, Jong-Min Lee

**Affiliations:** ^1^Division in Anatomy and Developmental Biology, Department of Oral Biology, Oral Science Research Center, BK21 PLUS Project, Yonsei University College of Dentistry, Seoul, South Korea; ^2^Applied Oral Sciences, Faculty of Dentistry, The University of Hong Kong, Hong Kong, Hong Kong

**Keywords:** circumvallate papilla, LGR5, Fgf10, cell proliferation, apoptosis

## Abstract

Taste buds develop in different regions of the mammal oral cavity. Adult stem cells in various organs including the tongue papillae are marked by leucine-rich repeat-containing G protein-coupled receptor 5 (*Lgr5*) and its homolog, *Lgr6*. Recent studies have reported that adult taste stem/progenitor cells in circumvallate papilla (CVP) on the posterior tongue are *Lgr5*-positive. In this study, we confirm the *Lgr5* expression pattern during CVP development. A previous study reported that mesenchymal *Fgf10* is necessary for maintaining epithelial *Lgr5*-positive stem/progenitor cells. To confirm the interaction between *Lgr5*-positive CVP epithelium and mesenchymal factor FGF10, reverse recombination (180-degree) was performed after tongue epithelium detachment. FGF10 protein-soaked bead implantation was performed after reverse recombination to rescue CVP development. Moreover, we reduced mesenchymal *Fgf10* by BIO and SU5402 treatment which disrupted CVP morphogenesis. This study suggests that the crosstalk between epithelial *Lgr5* and mesenchymal *Fgf10* plays a pivotal role in CVP epithelium invagination during mouse tongue CVP development by maintaining *Lgr5*-positive stem/progenitor cells.

## Introduction

Taste buds develop in different regions of the oral cavity in mammals. Taste buds localize in three different types of taste papillae on the mammalian tongue. The anterior dorsum of the tongue is covered with fungiform papillae (FFPs). Circumvallate papillae (CVPs) are localized medially on the posterior portion of the tongue. Foliate papillae (FOPs) are located laterally on the posterior side of the tongue (Kist et al., 2014)

Based on functional and morphological studies, there are three types of mature taste bud cells, type 1 (glial-like cells), type 2 (responsible for sensing bitter, sweet and umami), and type 3 (sour sensors as presynaptic cells), as well as immature taste bud cells, which are type 4 (basal cells that are precursors of other types of mature taste cells) (Kapsimali and Barlow, 2013; Miura et al., 2014). Different taste bud subtypes may have different longevities (Beidler and Smallman, 1965; Hamamichi et al., 2006; Perea-Martinez et al., 2013; Mistretta and Kumari, 2017).

Epithelial thickening of CVP is first observed at E11.5 in the developing mouse tongue. At E13.5, CVP epithelium invagination occurs into the adjacent tongue mesenchyme. From E15.5 to E16.5, a “dome-like” structure of CVP is detected with epithelial stalks. Epithelial stalks of CVP invaginate more deeply into the underlying mesenchyme at E17.5 (Jitpukdeebodintra et al., 2002). Taste buds are localized in the trench wall epithelium of the CVP with specific patterning. However, the mechanisms of CVP epithelial invagination are not well understood. Previous studies have reported that specific gene expression patterns may regulate proper CVP morphogenesis and taste bud pattern formation (Jitpukdeebodintra et al., 2002; Lee et al., 2006; Kim et al., 2009).

Leucine-rich repeat-containing G protein-coupled receptor 5 (*Lgr5*) marks taste bud stem and progenitor cells in CVP. *Lgr5*-positive cells give rise to all major types of taste bud cells confirmed by *Lgr5*-EGFP-IRES-creERT2 mice (Takeda et al., 2013; Yee et al., 2013). Previous studies have reported that *Lgr5*-positive stem cells are localized in various organs such as the small intestine, colon, stomach, hair follicle, liver, pancreas, and cochlea (Barker et al., 2013). In addition, various organoids are generated by *Lgr5*-positive stem cells including taste bud organoid (Sato et al., 2009; Huch et al., 2013a,b; Ren et al., 2014). *Lgr5* marks an active stem cell population for taste buds in the adult mouse CVP, localized in the posterior part of the tongue (Ren et al., 2014). However, the *Lgr5* expression pattern during CVP development is still unknown. Lgr6, as an *Lgr5* homolog, also marks taste stem and progenitor cells in taste buds (Hevezi et al., 2009). Lgr6 is expressed in FFP and CVP taste buds as a taste stem/progenitor cell marker (Zhao et al., 2011).

The expression pattern of various Fibroblast Growth Factors (FGFs) and their receptors has been elucidated in the developing tongue (Nie, 2005; Sohn et al., 2011; Du et al., 2016). *Fgf10* is strongly expressed in the developing CVP mesenchyme, and *Fgf10* knockout (KO) mice show a loss of the CVP phenotype (Petersen et al., 2011). One of the key regulators of the epithelial stem cells in the mouse incisors is mesenchymal *Fgf10*. After BIO, Wnt activator treatment, mesenchymal *Fgf10* is dramatically reduced and loss of epithelial *Lgr5* is observed in mouse incisor. For the maintenance of *Lgr5*-positive epithelial stem cells in the apical bud, *Fgf10* is required in the surrounding mesenchyme (Yang et al., 2015).

Here, we hypothesized that crosstalk between epithelial *Lgr5* and mesenchymal *Fgf10* signaling may be required for CVP formation, especially in epithelial invagination. We found that FGF10 was localized in the CVP mesenchyme at E15.5 and E17.5 but not in the anterior FFP-forming region. After a reverse recombination assay (180-degree), the rotated tongue CVP epithelium had an FFP-like structure without epithelial invagination. FGF10-soaked beads implanted after reverse recombination could rescue CVP morphogenesis. After BIO, WNT activator, and SU5402, FGF signaling inhibitor, treatment, the expression levels of *Lgr5* was reduced, and CVP morphology was disrupted. Our results suggest that CVP epithelial invagination may require mesenchymal *Fgf10*.

## Materials and Methods

All experiments were performed according to the guidelines of the Yonsei University College of Dentistry, Intramural Animal Use and Care Committee.

### Animals

Adult Institute of Cancer Research; Caesarian Derived-1 (ICR; CD-1) mice were housed in a temperature-controlled room (22°C) under artificial illumination (lights on from 05:00 to 17:00) and 55% relative humidity. The mice had access to food and water *ad libitum*. Embryos were obtained from time-mated pregnant mice. E0 was designated as the day a vaginal plug was confirmed. Embryos at developmental stages E13.5, E15.5, E17.5, PN1, and adult mice were used in this study.

### *In vitro* Organ Culture

The developing tongue was isolated from E15.5 mouse and cultured on 1.0 μm Nucleapore Track-Etch Membrane (Whatman, United States) in medium at 37°C and 5% CO_2_ for 72 h using a slight modification of the culture method reported by Trowell (Kim et al., 2009). The culture medium (DMEM/F12, Invitrogen, United States) was supplemented with 2 mM GlutaMAX (Invitrogen, United States), 10 mM HEPES buffer (Sigma-Aldrich, United States), 2% B27 (Invitrogen, United States) and 1% penicillin/streptomycin and was renewed every 24 h.

### Recombination Assay

Recombination of the tongue using the intact epithelium and mesenchyme was done at E15.5. From E15.5, CVP is clearly recognized under microscope. The tongue was dissected and approximately 0.05 ml of Indian ink (Royal Talens, Holland) was injected using a 25-gage needle into CVP regions. Dissected tongues were incubated in 2.4 unit Dispase II (neutral protease, grade II) (Roche Applied Science, Switzerland) for 30 to 50 min at 37 °C, and washed in medium containing 10% fetal bovine serum. The epithelium and mesenchyme were separated on ice under a dissection microscope. Separated epithelia remained intact in the media. The epithelium was placed on top of the mesenchyme with a 180-degree rotation, from anterior to posterior, and the recombinants were cultured for 72 h using Trowell’s method.

### Histology and Immunohistochemistry

Samples were fixed in 4% paraformaldehyde in phosphate buffered saline (PBS) and then embedded in paraffin using standard procedures. Serial paraffin sections (4-μm thickness) were prepared for Hematoxylin and Eosin (HE), immunostaining and *in situ* hybridization. Antigen retrieval was achieved by citrate buffer, pH 6.0. After antigen retrieval, immunohistochemical analyses were performed using the DakoCytomation Envision System (using horseradish peroxidase with diaminobenzidine enhancer) (Dako, United States) according to the manufacturer’s instructions. The slides were incubated with antibodies against Pan-cytokeratin (Pan-CK) (1:50, Thermo Fisher Scientific^TM^, United States), FGF10 (1:50, Santa Cruz, Unite States), Caspase 3 (1:1600, Cell Signaling Technology, United States) and Ki67 (1:200, Abcam, United Kingdom). The specimens were sequentially incubated with secondary antibody and streptavidin peroxidase. Finally, the results were visualized following staining using a diaminobenzidine reagent kit (Invitrogen, United States). The sections were counterstained with hematoxylin. All specimens were observed by stereomicroscope (MD5500D; Leica, camera: DFC495; Leica, Lens: HCX PL APO 409; Leica). At least 10 mice were examined in each experiment.

### *In situ* Hybridization

All samples were fixed in 4% paraformaldehyde in PBS and 4 μm thickness paraffin sections under RNase free circumstance were prepared for section *in situ* hybridization. *In situ* hybridization for *Lgr5* was performed using RNAscope 2.5 Assay (Advanced Cell Diagnostics, ACD, United States) according to manufacturer’s protocols (Wang et al., 2012). RNAscope *Lgr5* probes were designed and validated by ACD. Paraffin sections were deparaffinized and heated in boiling target retrieval buffer and pretreated with protease prior to hybridization with target oligo probes. Color development was performed with Fast Red substrate. Intracellular red punctate dots are considered as positive signal.

### Bead Implantation

Heparin formate-derived beads (Sigma-Aldrich, United States) were incubated in 50 μg/ml recombinant human FGF10 (R&D systems, United States) for 1 h at room temperature. FGF10-soaked beads were implanted between Indian ink labeled CVP epithelium and tongue mesenchyme at E15.5 and cultured for 72 h. From E15.5, CVP is clearly recognized under microscope. 0.1% BSA in PBS-soaked beads were implanted as controls.

### Inhibition of FGF Signaling

For inhibition of FGF signaling on the cultured developing tongue, DMSO (Millipore), SU5402 (Calbiochem, Germany), and BIO (Sigma-Aldrich, United States) were used. The developing tongues were dissected at E15.5 in PBS and cultured using Trowell’s method for 72 h with media containing 20 μm SU5402 or 1 ng/ml BIO. DMSO was used for control.

### RNA Preparation and Real-Time Quantitative Polymerase Chain Reaction Analysis (RT-qPCR)

Total RNA of cells were extracted using Trizol reagent. The extracts were reverse transcribed using Maxime RT PreMix (#25081; iNtRON, Korea). RT-qPCR primer sets designed using Primer Express software (Applied BiosyStems, United States) and RT-qPCR was performed using StepOnePlus Real-Time PCR System (Applied BioSystems, United States). The amplification program consisted of 40 cycles of denaturation at 95°C for 15 s, annealing at 60°C for 1 min and extension at 72°C for 20s. The expression levels of each gene are expressed as normalized ratios against the GAPDH housekeeping gene. The oligonucleotide RT-PCR primers for *Fgf10*, *Lgr5*, and Glyceraldehyde-3-phosphate dehydrogenase (GAPDH) are as follows:

*Lgr5*-Foward: AGC ATG CTT CTG GCA AGA TGT TC

*Lgr5*-Reverse: GAC TTA ACG CCC TGC GTT TGA

*Fgf10*-Foward: CAT CTG CGG AGC TAC AAT CA

*Fgf10*-Reverse: CCC CTT CTT GTT CAT GGC TA

*GAPDH*-Foward: GTCATCATCTCCGCCCCTTCTG

*GAPDH*-Reverse: ATGCCTGCTTCACCACCTTCTTG

### Quantification of Ki67 and Caspase3-Positive Cells

Ten slides that contained five sections each were used to determine the number of Ki67 and Caspase3-positive cells, and 15 sections were chosen at random from the 10 slides. The number of cells was counted in an area of 100 × 100 μm. After counting the proliferating cells, we corrected the counting results using Abercrombie’s method (Abercrombie, 1946).

The equations used is

$$P=A\frac{M}{L+M}$$

*P* is the average number of nuclear points per section, A the crude count of number of nuclei seen in the section, M the thickness of the section and L is the average length of the nuclei. Data were expressed as the mean ± *SD*.

## Results

### Expression Pattern of *Lgr5* During Mouse CVP Development

**FIGURE 1:**
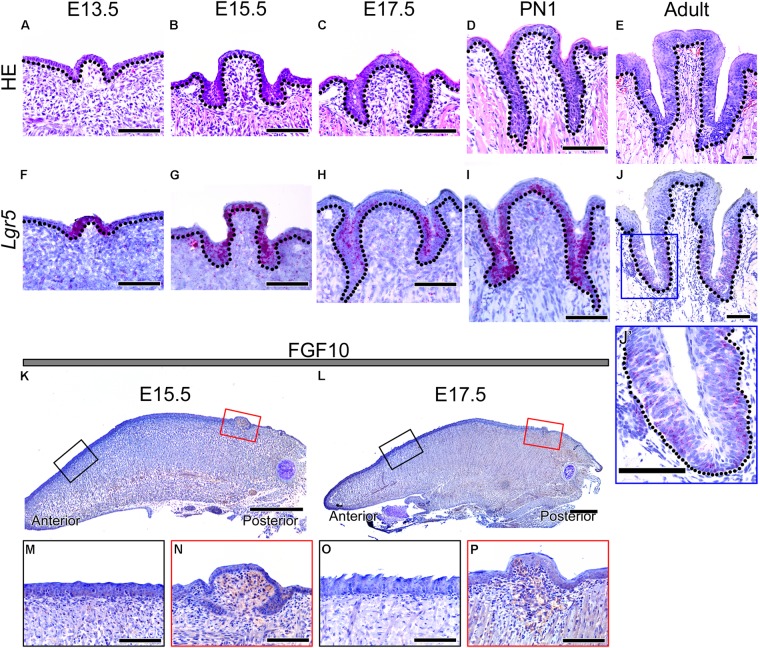
Morphology of developing CVP and expression patterns of *Lgr5* and FGF10 during CVP development. **(A–E)** HE staining shows the dynamic morphological changes during CVP development. **(A)** The epithelial invagination of CVP into the adjacent mesenchyme was observed at E13.5. **(B)** At 15.5, the epithelial invagination of both sides of CVP had deepened. **(C)** At 17.5, deep trenches were formed from the epithelium invagination of CVP. **(D)** At PN1, von Ebner’s glands were connected to the invaginated epithelium of CVPs. **(E)** At the adult stage, circular furrows and taste buds were observed in the well-formed epithelial trenches of CVP. **(F–J, J’)** The *Lgr5* expression pattern was confirmed by *in situ* hybridization. **(F)** *Lgr5* expression was observed in the CVP epithelium but not in the tongue epithelium or adjacent mesenchyme at E13.5. **(G)** At 15.5, *Lgr5* was strongly expressed in the epithelial invagination region and dorsal epithelium of CVP. **(H)** At 17.5, strong *Lgr5* expression was detected in the epithelial trenches of CVP but partially observed in the dorsal epithelium of CVP. **(I)** Expression of *Lgr5* was detected in the taste bud-forming region but not in the circular furrow-forming region, while partial expression was detected in the dorsal epithelium of CVP at PN1. **(J)** In the adult stage, the expression was only detected around taste buds and bottom region of the epithelial trench. **(J’)** Higher magnification of the blue box in Panel **J**. **(K,L)** At 15.5 and 17.5, FGF10 was strongly localized in the mesenchyme of the CVP-forming region but not in the FFP-forming region. **(M–P)** Higher magnification results indicated that the localization of FGF10 was strongly detected in the CVP mesenchyme but not in the FFP mesenchyme at E15.5 and at 17.5. Scale bars are 100 μm in **(A–J,J’,M–P)** and 500 μm in **(K,L)**.

To confirm the morphology of developing CVP, HE staining was performed from E13.5 to adult (**Figures 1A–E**). CVP epithelial invagination into the adjacent mesenchyme was observed at E13.5 (**Figure 1A**). Deeper CVP trenches were detected on both sides of the CVP at E15.5 (**Figure 1B**). CVP epithelium formed deep trenches, called epithelial crypts, at E17.5 (**Figure 1C**). CVP epithelial invagination formed deeper trenches, and these were connected to Von Ebner’s glands at PN1 (**Figure 1D**). Adult CVP showed well-formed epithelial trenches with circular furrows and taste buds through CVP epithelial trenches (**Figure 1E**).

To confirm the localization of *Lgr5*, *in situ* hybridization was performed during CVP development (**Figures 1F–J,J**’). *Lgr5* was expressed in the CVP epithelium but not in the tongue epithelium or adjacent mesenchyme at E13.5 (**Figure 1F**). AT E15.5, *Lgr5*-positive cells were detected in the epithelial invaginated trench region and in the dorsal epithelium of CVP (**Figure 1G**). At E17.5, strong *Lgr5* expression was observed in the invaginated CVP epithelium but partially detected in the dorsal surface of the epithelium (**Figure 1H**). *Lgr5* expression was detected in the taste bud-forming region except for the circular furrow-forming region at PN1. Moreover, *Lgr5* was partially expressed in the dorsal epithelium of CVP (**Figure 1I**). In the adult, *Lgr5* was expressed in taste buds and the bottom region of the epithelial trenches (**Figures 1J,J**’).

### FGF10 Localized to the Developing CVP Mesenchyme

To confirm the localization of FGF10 in developing CVP, immunohistochemistry (IHC) was performed at E15.5 and E17.5, which are critical stages of CVP epithelial invagination. To compare the mesenchyme of the FFP- and CVP-forming region, we used sagittal sections of the developing tongue (**Figures 1K–P**). FGF10 was strongly localized in the CVP mesenchyme but not in the FFP-forming anterior mesenchyme at E15.5 (**Figure 1K**). Higher magnification clearly indicated that FGF10 was detected in the CVP mesenchyme but not the FFP mesenchyme (**Figures 1M,N**). At E17.5, FGF10 was also only detected in the CVP mesenchyme (**Figure 1L**). Higher magnification showed that FGF10 was strongly localized to the CVP mesenchyme but not the FFP mesenchyme (**Figures 1O,P**).

### Morphological Analysis After Reverse Recombination of the Tongue Epithelium

**FIGURE 2:**
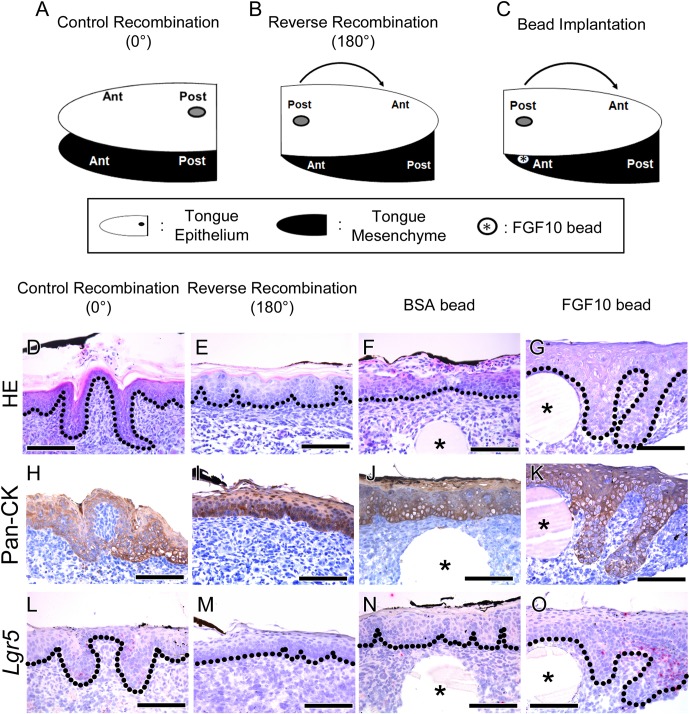
Morphological differences and rescue study after tongue epithelium recombination. **(A–C)** Schematic diagram indicating the three different experiments for *in vitro* culture. **(A)** In the control recombination, the CVP epithelium was separated, recombined in the same position and cultured for 72 h. **(B)** In the reverse recombination condition, the tongue epithelium was rotated 180 degrees in the anterior-posterior axis and then recombined with the tongue mesenchyme. To indicate the CVP region, Indian ink marks the dorsal surface of the CVP epithelium. **(C)** FGF10-soaked bead implantation was performed under the Indian ink-labeled CVP epithelium in the reverse recombination condition. **(D)** HE staining shows proper morphogenesis of the developing CVP in the control recombination condition (*n* = 9/10). **(E)** In the reverse recombination tongue, FFP-like structure was observed in the CVP epithelium (20/20). **(F,G)** Implantation of FGF10-soaked beads rescued CVP epithelial invagination in the reverse recombination condition, while BSA-soaked beads could not rescue CVP morphology. **(H,I)** Epithelial cell marker Pan-CK was clearly localized in epithelium of control and reverse recombination. **(J,K)** Pan-CK was strongly localized both in BSA and FGF10 bead implanted group. **(L–O)** *Lgr5* was expressed in control recombination and FGF10 bead implanted group. However, *Lgr5* signal was negative in reverse recombination and BSA bead implanted group without epithelial invagination. ^∗^, bead. Scale bars are 100 μm.

To determine region-specific tongue papillae morphogenesis, reverse recombination of the E15.5 mouse tongue epithelium was performed (**Figure 2**). The schematic diagram shows the three different experimental designs for the recombination of the tongue epithelium. In the control recombination condition, the tongue epithelium was separated with Dispase II, recombined in the same position in relation to the tongue mesenchyme and cultured for 72 h (**Figure 2A**). Reverse recombination indicated that the separated tongue epithelium was turned 180 degrees, from anterior to posterior, and then recombined with the tongue mesenchyme (**Figure 2B**). To confirm the CVP region, Indian ink labeling was performed on the CVP dorsal surface before separation of the tongue epithelium. To determine the function of mesenchymal FGF10, FGF10-soaked bead implantation was performed in the CVP region of the reverse recombination condition (**Figure 1C**).

Hematoxylin and Eosin staining results indicated that CVP developed properly in the control recombination group (*n* = 9/10) (**Figure 2D**). However, CVP structure was not detected; rather, an FFP-like structure was observed in the CVP epithelium region after reverse recombination (*n* = 20/20) (**Figure 2E**). To rescue CVP epithelial morphogenesis, FGF10-soaked bead was implanted in the reverse recombination developing tongue. Implanted FGF10-soaked beads rescued the epithelial invagination of CVP after reverse recombination (9/15) (**Figure 2G**). However, 0.1% BSA-soaked control bead could not rescue CVP morphology (10/10) (**Figure 2F**).

To confirm the CVP epithelium in recombination and bead implantation group, IHC was performed using Pan-CK antibody. Strong Pan-CK localization was detected in both control, reverse recombination (**Figures 2H,I**). Epithelial invagination of CVP was not observed in reverse recombination group (**Figure 2I**). BSA bead implanted specimen showed similar epithelial morphology as reverse recombination group (**Figure 2J**). FGF10 bead implanted CVP epithelium clearly showed that rescuing epithelial invagination with Pan-CK (**Figure 2K**).

To confirm the relationship between *Lgr5* expression and CVP epithelial invagination, *in situ* hybridization was performed using *Lgr5* probe. *Lgr5*-positive cells were observed at epithelial trench region in control recombination (**Figure 2L**). However, *Lgr5*-positive cell and epithelial invagination was not detected in reverse recombination group (**Figure 2M**). BSA bead was not rescue epithelial invagination and *Lgr5*-positive cell was not detected (**Figure 2N**). FGF10 bead clearly rescue CVP epithelial invagination and *Lgr5*-positive cells were observed in CVP epithelium (**Figure 2O**).

### Disruption of CVP Morphogenesis by Inhibiting *Fgf10* Signaling

**FIGURE 3:**
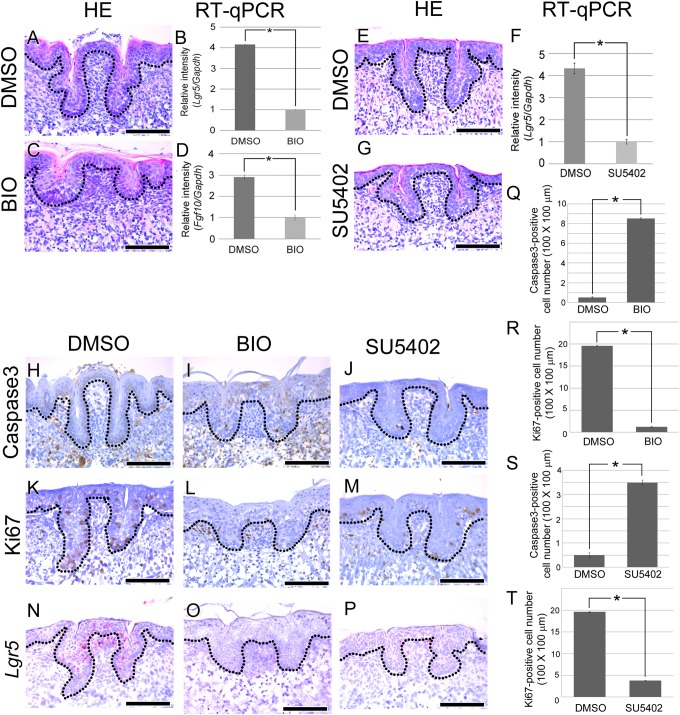
Morphogenesis and cellular events analysis after BIO treatment. **(A–T)** E15.5 mouse tongues were treated with BIO (1 ng/ml) or SU5402 (20 μm) and cultured for 72 h. **(A)** In the control group, normal CVP morphology was observed. **(C)** In the BIO-treated group, significantly disrupted CVP structure was observed with a reduced epithelial invagination depth. **(B,D)** To confirm the efficiency of BIO treatment, RT-qPCR was performed. *Fgf10* and *Lgr5* expression levels were significantly reduced after BIO treatment compared to those in the control group. **(E,G)** CVP morphology was disrupted by SU5402 treatment. **(F)** RT-qPCR result indicated that *Lgr5* expression level was reduced after SU5402 treatment. **(H)** No Caspase3-positive CVP epithelial cells were detected in the control group. **(I,J)** In the BIO and SU5402 treatment group, apoptotic cells were detected at the crypt region of disrupted CVP epithelium where stem cells are normally located. **(K)** Numerous Ki67-positive proliferating cells were observed in not only the CVP epithelium but also the tongue epithelium. **(L,M)** Proliferating cells were reduced after BIO or SU5402 treatment both in the CVP epithelium and tongue epithelium. **(N)** *Lgr5* was expressed in DMSO control group. **(O,P)** Positive signal of *Lgr5* was not observed after BIO and SU5402 treatment. **(Q–T)** Apoptotic and proliferating cell number was counted after BIO and SU5402 treatment. Number of apoptotic cell was induced and proliferating cell was reduced by BIO and SU5402 treatment. Scale bars are 100 μm. ^∗^*p* < 0.05.

A previous study reported that mesenchymal *Fgf10* is necessary for maintaining epithelial *Lgr5*-positive stem/progenitor cells in the developing mouse incisor (Yang et al., 2015). Yang et al. (2015) have reported that BIO treatment reduce mesenchymal *Fgf10* during mouse incisor development. Moreover, reduced survival of epithelial *Lgr5*-positive stem/progenitor cells is observed when mesenchymal *Fgf10* decreased. CVP showed similar expression of mesenchymal FGF10 and epithelial *Lgr5* to mouse incisor (**Figure 1**). To determine whether CVP morphogenesis is regulated by *Fgf10* and *Lgr5*, BIO treatment was performed to the mouse tongue at E15.5 (**Figures 3A–L**). HE staining results showed that well-developed CVP was formed in the DMSO control group (*n* = 10/10) (**Figure 3A**). However, CVP morphology was disrupted after BIO treatment (*n* = 20/20) (**Figure 3C**).

To confirm the activity of BIO, real time-quantitative polymerase chain reaction (RT-qPCR) was performed. After BIO treatment, the expression levels of *Fgf10* and *Lgr5* were significantly reduced in the developing tongue (**Figures 3B,D**). To reveal the cause of CVP morphogenesis disruption, apoptosis was investigated using Caspase3. Caspase3-positive apoptotic cells were not detected in the CVP epithelium of the DMSO-treated control group (**Figure 3H**). However, apoptotic cells were observed in the disrupted CVP epithelium after BIO treatment (**Figure 3I**). To confirm the cell proliferation after BIO treatment, IHC was performed with Ki67. KI67-positive cells were observed in the basement membrane of the tongue epithelium, including in CVP (**Figure 3K**). Cell proliferation was dramatically reduced after BIO treatment both in the tongue epithelium and CVP epithelium (**Figure 3L**).

To reveal the relationship between mesenchymal FGF signaling and epithelial *Lgr5* expression, *in situ* hybridization of *Lgr5* was performed after BIO treatment. In DMSO control, Lgr5 was strongly expressed in CVP epithelium (**Figure 3N**). However, Lgr5-positive signal was not observed in disrupted CVP epithelium after BIO treatment (**Figure 3O**). Number of apoptotic cell and proliferating cell were counted after BIO treatment. Apoptotic cell number was increased after BIO treatment compared to DMSO treated control group (**Figure 2Q**). However, Ki67-positive proliferating cells were decreased in BIO treated group (**Figure 2R**).

To confirm direct effect of FGF signaling to CVP morphogenesis via *Lgr5*, SU5402 was treated during CVP development. After SU5402 treatment, CVP morphology was disrupted (**Figures 3E,G**). To confirm the *Lgr5* expression level after inhibition of FGF signaling, RT-qPCR was performed. *Lgr5* expression level was significantly reduced after SU5402 treatment compared to DMSO treated control (**Figure 3F**). Caspase3-positive apoptotic cells were observed in SU5402 treated group compared to DMSO control (**Figure 3J**). In SU5402 treated group, reduced proliferating cell was confirmed by Ki67 (**Figure 3M**). *Lgr5* expression also reduced after SU5402 treatment in disrupted CVP region (**Figure 3P**). Number of apoptotic and proliferating cell were similar in BIO and SU5402 treated group. After SU5402 treatment, apoptotic cell number was increased but number of proliferating cell was reduced (**Figures 3S,T**).

## Discussion

Lingual papillae consist of four kinds of papillae: FFPs, filiform papillae, FOPs, and CVPs (vallate papillae), which all localize to the surface of the tongue. Except for filiform papillae, taste papillae contain taste buds. All types of lingual papillae have a specific region on the tongue in which they are formed; the CVP is localized to the posterior region of the tongue (Norton and Netter, 2012).

Epithelial invagination is a key factor for proper CVP morphogenesis (Jitpukdeebodintra et al., 2002). Epithelial invagination is observed in various organs such as the tooth, ear, and eye, and epithelial mesenchymal interaction is necessary for this complex molecular and cellular event (Pearl et al., 2017).

Previous studies have reported that stem/progenitor cell marker, *Lgr5*, and *Lgr6*, are expressed in developing CVP, but *Lgr5* is not expressed or instantaneously expressed in FFPs (Ren et al., 2014). In this study, the *Lgr5* expression pattern was revealed using *in situ* hybridization in CVP. In the early developmental stage of the tongue, *Lgr5* was expressed in the developing CVP epithelium (**Figures 1F,G**). In the late developmental stage, *Lgr5* was partially expressed in the CVP epithelium, especially in the taste bud-forming region (**Figures 1H,I**). The *Lgr5* expression region was restricted in the deep trench region of the CVP epithelium in the adult (**Figure 1J**). These results indicated that *Lgr5* related to epithelial invagination of CVPs and taste bud formation.

Previous studies have reported that *Fgf10* is expressed in the tongue mesenchyme just beneath the CVP epithelium. *Fgf10* KO mice show loss of CVPs in the tongue (Petersen et al., 2011). To compare FGF10 localization between the FFP- and CVP-forming region, IHC was performed using sagittal sections of the developing tongue. At E15.5 and 1.7.5, FGF10 was localized to the mesenchyme under CVP but not in the FFP-forming region (**Figures 1K–P**). These results indicate that mesenchymal FGF10 signaling may play a pivotal role in CVP development.

Mesenchymal expression of *Fgf10* is necessary for maintaining epithelial *Lgr5*-positive cells by inhibiting apoptosis in the mouse incisor (Yang et al., 2015). CVP also express epithelial *Lgr5*-positive cells and mesenchymal FGF10 during tongue development (**Figure 1**). To examine the role of mesenchymal FGF10, reverse recombination of epithelium was performed using the E15.5 mouse tongue (**Figure 2**). Indian ink-labeled CVP epithelium did not exhibit epithelial invagination on FGF10-negative tongue mesenchyme (**Figure 2E**).

To confirm that FGF10 could rescue CVP morphogenesis in reverse recombination, FGF10-soaked bead was implanted between the CVP epithelium and anterior mesenchyme. Epithelial invagination of CVP was rescued around the FGF10-soaked bead (**Figures 2F,G**). These results indicate that mesenchymal FGF10 plays a pivotal role in proper CVP morphogenesis, especially epithelial invagination.

BIO, a glycogen synthase kinase 3 inhibitor, activates Wnt signaling by preventing the degradation of β-catenin (Hedgepeth et al., 1997). In the developing lung and the lacrimal gland, canonical Wnt signaling can cause a decrease in *Fgf10* expression, which interrupt branching morphogenesis by inhibits cell proliferation (Dean et al., 2005). To normal peripheral taste development, Wnt/β-catenin signaling pathways are necessary and *Lgr5* is a Wnt target gene in taste cell (Iwatsuki et al., 2007; Ren et al., 2014). After BIO treatment, *Lgr5*-positive stem/progenitor cells are reduced by inhibiting the anti-apoptotic effect of *Fgf10* in the mouse incisor (Yang et al., 2015). To confirm the effect of *Fgf10* in the developing mouse tongue CVP, the mouse tongue was treated with BIO at E15.5. After BIO treatment, CVP morphology was destroyed. RT-qPCR results indicated that the *Lgr5* and *Fgf10* expression levels were reduced after BIO treatment. Moreover, the number of proliferating cells was reduced, and the number of apoptotic cells was increased by BIO treatment (**Figure 3**). These results indicated that BIO treatment affected to CVP morphogenesis by abnormal Wnt signaling and *Fgf10* expression.

To reveal the relationship between mesenchymal FGF signaling and epithelial *Lgr5*, SU5402, FGF signaling inhibitor, was treated during CVP development. Inhibition of FGF signaling by SU5402 induce similar effect as BIO treatment. CVP morphology was disrupted by induced apoptosis and reduced cell proliferation. Moreover, Lgr5 expression was markedly reduced in CVP epithelium (**Figure 3**). These results indicated that mesenchymal *Fgf10* plays an important role in CVP morphogenesis through an anti-apoptotic effect on epithelial *Lgr5*-positive cells during CVP development.

**FIGURE 4:**
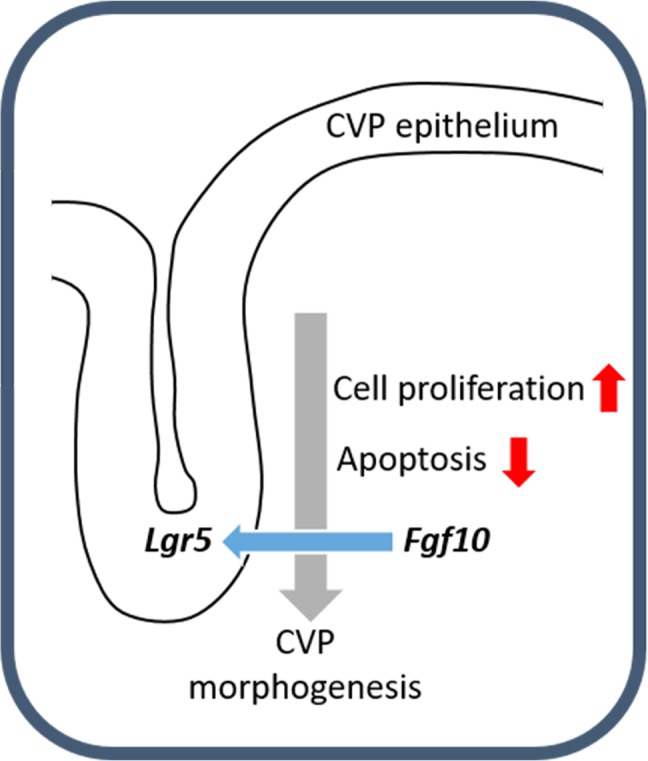
Schematic representation of CVP development. The molecular interactions between *Fgf10* and *Lgr5* modulate cellular events including apoptosis and cell proliferation to ensure correct CVP development.

In summary, *Lgr5* was expressed in the CVP epithelium during CVP development. FGF10 was localized in the tongue mesenchyme just beneath the CVP-forming region. When the developing CVP epithelium was recombined to the anterior mesenchyme, in the absence of the mesenchymal FGF10 region, CVP structure was not observed. Importantly, FGF10-soaked bead could rescue CVP morphogenesis. After BIO or SU5402 treatment, abnormal apoptosis and cell proliferation were detected in the CVP epithelium by reducing epithelial *Lgr5* expression (**Figure 4**). These results indicate that crosstalk between epithelial *Lgr5* and mesenchymal *Fgf10* is necessary for proper CVP morphogenesis during tongue development.

## Ethics Statement

Institutional Animal Care and Use Committee Yonsei University Health System (IACUC). IACUC Approval No. 2015-0333. Title of Protocol: Study on function of Lgr5-positive cells in murine oral regenerative tissues.

## Author Contributions

SZ and HSC performed *in situ* hybridization, tongue culture, IHC, and RT-qPCR. H-SJ and J-ML contributed to the design and implementation of the research, to the analysis of the results and to the writing of the manuscript.

## Conflict of Interest Statement

The authors declare that the research was conducted in the absence of any commercial or financial relationships that could be construed as a potential conflict of interest.
